# Sources of negative emotions and tactics of self-emotion regulation among college students during COVID-19 school closure in China

**DOI:** 10.3389/fpubh.2024.1265350

**Published:** 2024-03-20

**Authors:** Hai Fu, Mingfeng Pan, Mingdong Lai

**Affiliations:** ^1^School of Liberal Arts, Nantong University, Nantong, Jiangsu, China; ^2^School of Education Science, Nantong University, Nantong, Jiangsu, China; ^3^School of Humanities and Media, Ningbo University, Ningbo, Zhejiang, China

**Keywords:** new coronary pneumonia epidemic situation, college students, negative emotions, emotional regulation strategies, COVID-19

## Abstract

**Background:**

This study investigated the level of anxiety and depression in Chinese college students since the COVID-19 pandemic and explored the sources of their negative emotions and students' self-emotion regulation strategies.

**Methods:**

A stratified cluster sampling questionnaire was used to survey college students during the pandemic via the Anxiety Depression, Self-made Negative Emotion Source, and Negative Emotion Regulation Strategy Scales.

**Results:**

The prevalence of anxiety and depression was 23.3 and 20.1%, respectively. These levels were higher in women than in men. Furthermore, senior students reported higher levels than freshmen. Anxiety and depression mainly came from the pressure to grow and the narrowed scope of social activities. Proper relaxation via entertainment and communication with family and friends were popular ways of regulating their negative emotions.

**Conclusion:**

College students should confront their negative emotions and understand their source, use psychological methods to regulate their anxiety and depression or seek professional help, improve their psychological resilience, and adopt positive coping measures.

## 1 Introduction

From the Black Death, Justinian plague, and Spanish flu to severe acute respiratory syndrome (SARS), the history of mankind has been shadowed by outbreaks (1). Humans experience physical and mental health issues with each outbreak. Maladaptive behaviors, emotional distress, and defensive responses are the main adverse psychological reactions that people tend to have in response to a disease pandemic (2). COVID-19 erupted globally in 2019, and scholars worldwide conducted abundant research on the psychological conditions of people during this pandemic. Previous research found that psychological distress and various public health emergencies emerged (3). People were easily engrossed in the flood of information and swamped by concerns, which could result in depression, anxiety disorders, substance abuse, increased suicidal tendencies, and post-traumatic stress disorder (4). Furthermore, quarantine measures that limited interpersonal contact substantially impacted people's mental health (5). People in quarantine were separated from relatives and friends, confronted with the loss of freedom, uncertainty over the disease status, and boredom (6). Children and college students were trapped at home and unable to receive regular education, which resulted in anxious, depressed, fatigued, and distressed feelings (7). Hence, governments, hospitals, educational institutions, organizations, and individuals should consider psychological interventions and adopt the necessary measures (8). Entrepreneurial activity is enterprising human action in pursuit of the generation of value through the creation or expansion of economic activity by identifying and exploiting new products, processes, or markets (9). Entrepreneurial activities were also threatened by the pandemic, as survival became extremely challenging without support and relief from the respective countries' governments and their policies (10). A study demonstrated a positive effect of several dimensions of emotional intelligence (EI) on cognition as a mediator (11). Hence, our worldview of a phenomenon could limit the most substantial options to understand and respond to unfavorable global situations (12).

In China, many universities took various control measures to prevent the transmission of COVID-19 and improve students' sanitary conditions, such as closed campuses, transitioning to online classes, raising medical supplies, providing psychological assistance, controlling personnel flow, and innovative teaching (13). During an outbreak, universities in the epicenter accommodated their students on campus. During the lockdowns, students could only interact with their roommates, and the scope of activities was relatively limited. Despite Chinese college students being heavily exposed to mental pressure, limited research focused on students' mental health during this period of school closure in China.

According to experiences in global pandemic prevention, the formulation and implementation of mental health assessments, support, treatment, and service are vital and urgent goals of the health response to COVID-19 (14). Furthermore, considering the protracted nature of COVID-19, the assessment of psychiatric distress and coping mechanisms is critical (15). College students' mental health should be examined during the closed school period. Studies should examine the damaging emotional crises among college students and ways to resolve them.

Previous studies indicated that emotion regulation processes were fundamental to normal and abnormal functioning (16). Emotion management refers to individuals' ability and processes to understand and control their emotions and deal with others' emotions (17). From the perspective of functionalism, self-emotion management is a process of improving individual self-emotion to adapt to different situations and tasks (18).

With the emergence of the ongoing novel COVID-19 pandemic, students in higher education experienced increased mental health challenges (19). Rapid changes in education were inevitable owing to changes in clinical settings and the impact of the repeated outbreaks (20). Further efforts are required to prevent adverse mental health outcomes and reduce the prevalence of mental health problems (21). Clinical nurses' negative psychology positively impacted burnout. Furthermore, emotional intelligence and self-efficacy in emotion management alleviated the influence of negative psychology on burnout (22). Significant associations were observed between all the dimensions of personality traits, emotional stability, and mental health (23). For college students, regulating emotions could help them protect against changes in depression and affect the quality of their social interactions (24). Therefore, this study established the following research questions.

According to the stress theory, after a stressful event occurs, the public will inevitably experience negative emotions. This was because emotions were an advanced functional pattern that could coordinate physiological, cognitive, motivational, behavioral, and subjective responses and increase human adaptability to threats and changes in the surrounding environment (25). Closed management of universities was undoubtedly an adverse life event for most college students. It had a severe impact on students' mental health. Hence, it was significant to explore the specific negative emotions that college students generated. Therefore, we established question 1 as follows: What were the negative emotions of Chinese college students during the closure period?

Bandura proposed the theory of ternary interactive determinism, which viewed the environment, behavior, and individual as relatively independent theoretical entities that interacted with and determined each other. Among them, the individual as the subject included their physiological response ability, cognitive ability, and other physical and mental abilities. Interactive decision refers to the causal relationship between the environment, behavior, and people, each of which has a bi-directional interaction and decision-making relationship (26). In online public opinion and major disaster events, people were prone to negative emotions or pessimistic attitudes toward aggressive behavior under the influence of internal or external factors (27). However, the specific causes remain unknown. Hence, question 2 was: What were the primary sources of these negative emotions? What were the fears of disease, school work burden, or limited campus activities?

When in a state of negative emotions, individuals often self-regulated. Kirby promoted survival and development (28). Some studies suggested that emotional management referred to how individuals effectively guided and regulated their emotions to maintain a relatively stable emotional state through legitimate and reasonable methods when they experienced adverse events (29). College students often adopt different ways of self-regulation when they experience negative emotions. Hence, we proposed questions 3 and 4. Question 3: what measures have students taken to achieve self-emotion management and eliminate negative emotions? Question 4: What other measures could students take to achieve self-emotion management and eliminate negative emotions?

This study investigated the anxiety and depression levels of college students and explored the source of students' negative emotions and their self-emotion regulation strategies. Furthermore, this study also provided a theoretical basis and inspiration for college students to improve their mental health levels while colleges closed during the COVID-19 pandemic. Both domestic and international psychologists have conducted little research on the source of negative emotions and the self-emotion regulation of college students during the closure of schools. This study examined the source of negative emotions, such as anxiety and depression, through questionnaires. It also proposed strategies for college students' self-emotion management to provide reference methods.

## 2 Methods

### 2.1 Participants and procedures

The participants were 422 college students from colleges closed due to the pandemic, regardless of their grades. After the responses were checked and screened to exclude unqualified ones, 481 questionnaires were sent out, and 399 valid questionnaires were received (effective recovery = 82.95%).

We used a national online survey platform called Wenjuanxing. The questionnaire used unified guidelines to describe the survey's purpose and significance. One IP address could only answer the questionnaire once, which could only be submitted once all the items were completed. After the questionnaires were collected, researchers checked each questionnaire's response time and eliminated unqualified questionnaires via the site management background of the online survey platform (30).

### 2.2 Research tools

#### 2.2.1 General situation questionnaire

The self-developed general situation questionnaire included participants, sex, age, grade, experiences of the pandemic since 2020, most extended period of school closure (within 1 week, 1 week to half a month, half a month to 1 month, 1–2 months, 2–3 months, or the entire semester), and form of closure adopted by the school (only activities in the dormitory, free activities on campus, cannot leave school unless necessary, free activities on campus, or can be approved to leave the school).

#### 2.2.2 Self-rating depression anxiety scale

Clark and Watson (31) proposed a three-factor structural model to distinguish common emotional disorders, such as depression, anxiety, and stress. Lovibond et al. (32) developed the Depression Anxiety Stress Scale (DASS) based on this model. The DASS has been widely used to evaluate and distinguish between clinical and non-clinical emotional disorders. The DASS-21 was a simplified version and excluded the top seven items with the highest workload in each dimension (33). The Self-Rating Depression Anxiety Scale was selected from the DASS-21 and had high reliability and validity.

The Self-Rating Anxiety Depression Scale, which included two subscales and 14 items, was used to investigate individuals' experience of negative emotions, such as depression and anxiety. Responses were rated on a 4-point Likert scale that ranged from 0 (not consistent) to 3 (most or always consistent). The higher the score, the more serious the degree of depression or anxiety. A depression score of ≤9, 10–13, 14–20, 21–27, and >28 points was considered average, mild, moderate, severe, and extremely severe, respectively. Anxiety scores of ≤7, 8–9, 10–14, 15–19, and >20 points were considered standard, mild, moderate, severe, and extremely serious, respectively. The internal consistency coefficients of the two subscales were 0.77 and 0.79. Their construction reliability was between 0.72 and 0.80 (34).

## 3 Results

### 3.1 Statistical analysis of demographic variables

Table 1 presents the participants' characteristics. The number of participants in each university year (1st−4th year) was 100, 79, 51, and 169, respectively. Of these, 65.2% were female (*n* = 260). Among those who have had school closures since 2020, 244 (61.2%) had experienced closures for 2–3 months. Furthermore, 146 (36.6%) had experienced closure for the entire semester. In addition, the number of participants in free activities on campus was 28 (7.0%), 301 (75.4%) were free activities on campus, and 70 (17.5%) were free activities on campus.

**Table 1:** Statistical analysis of the demographic variables.

**Demographic variables**		***M* ±*SD***	***N* (%)**
Sex	Male	1.65 ± 0.48	139 (34.8)
	Female		260 (65.2)
Grade	Freshman	2.72 ± 1.25	100 (25.1)
	Sophomore		79 (19.8)
	Junior		51 (12.8)
	Senior		169 (42.4)
The longest lockdown time students experienced	Within a week	2.49 ± 1.46	20 (5.0)
	A week to half a month		52 (13.0)
	Half a month to a month		83 (20.8)
	Two to 3 months		98 (24.6)
	Entire semester		146 (36.6)
School closure form	Activities are in the dormitory only	1.93 ± 0.26	28 (7.0)
	Free activities on the campus, no leaving the campus unless necessary		301 (75.4)
	Free activities on the campus, being approved out of the school		70 (17.5)

### 3.2 Score of anxiety and depression in college students

**Figure 1:**
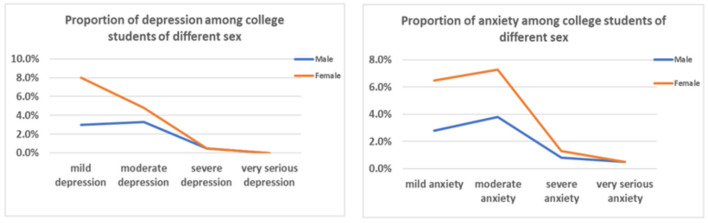
Comparison of the proportion of anxiety and depression among college students of different sexes.

**Figure 2:**
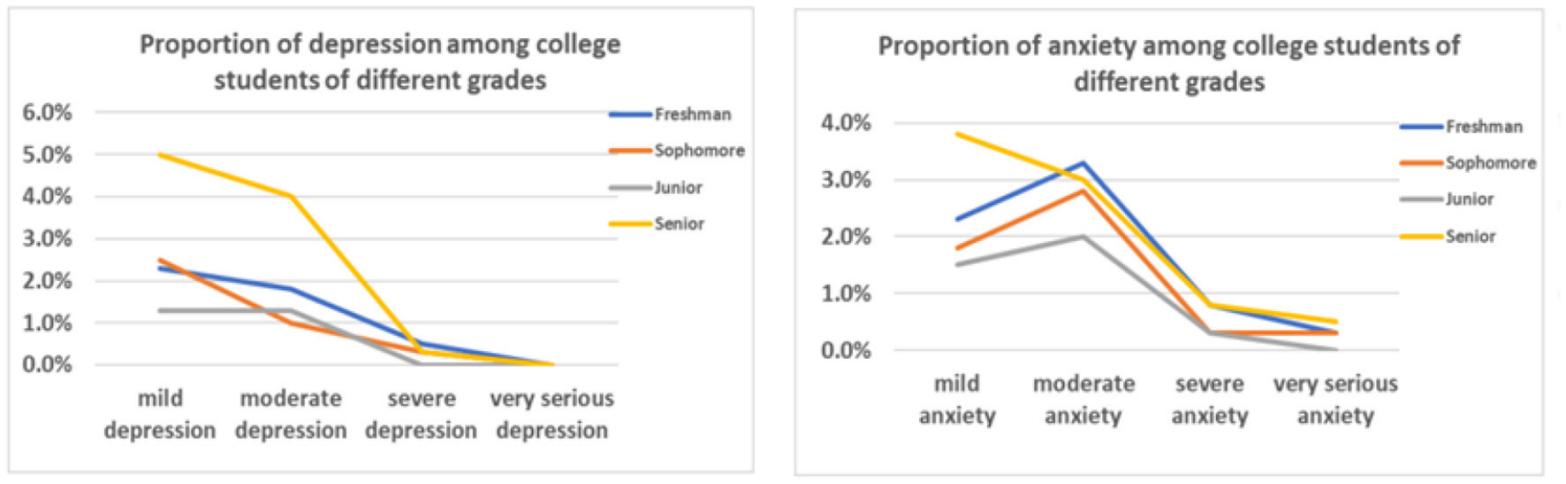
Comparison of the proportion of anxiety and depression among college students of different grades.

Table 2 presents the prevalence of anxiety and depression among college students during the closure period. The rates were 23.3 and 20.1%, respectively. Figure 1 presents the proportion of anxiety and depression among college students by sex. Results indicated that the levels of anxiety and depression were higher among women than among men. Figure 2 presents the proportion of anxiety and depression among college students by grade. Results indicated that the senior students had the highest levels of anxiety and depression. A univariate analysis of variance (ANOVA) was conducted and found a significant difference in the level of depression among college students in each grade [*F*_(3,397)_ = 2.931, *p* = 0.033]. Further analysis found a significant difference in the level of depression between freshmen and sophomores (*p* = 0.011) and a substantial difference between freshmen and seniors (*p* = 0.009; see Table 3). Spearman's rank correlation analysis showed that anxiety and depression were significantly correlated, *r*_(397)_ = 0.793, *p* < 0.01. This indicated that college students' anxiety and depression were highly positively correlated during school closures.

**Table 2:** Anxiety and depression levels in college students.

**Category**	**Anxiety**		**Depression**	
	**Number (*****n*** = **399)**	**Proportion/100%**	**Number (*****n*** = **399)**	**Proportion/100%**
Normal	306 (i ≤ 7)	76.7	319 (i ≤ 9)	79.9
Mild	37 (8–9)	9.3	44 (8–13)	11.0
Moderate	44 (10–14)	11.0	32 (14–20)	8.0
Severe	8 (15–19)	2.0	4 (21–27)	1.0
Extraordinary severe	4 (i > 20)	1.0	0 (i > 20)	0.0

**Table 3:** Descriptive statistics and analysis of variance (ANOVA) results of anxiety and depression among college students of different grades.

	**Depression**					**Anxiety**				
	***M* ±*SD***	**Freshman**	**Sophomore**	**Junior**	**Senior**	***M* ±*SD***	**Freshman**	**Sophomore**	**Junior**	**Senior**
Freshman	0.61 ± 0.64	1				0.48 ± 0.51	1			
Sophomore	0.88 ± 0.82	−0.27^*^	1			0.64 ± 0.62	−0.16^*^	1		
Junior	0.79 ± 0.60	−0.18	0.10	1		0.66 ± 0.51	−0.18^*^	−0.02	1	
Senior	0.84 ± 0.73	−0.24^**^	0.04	−0.06	1	0.52 ± 0.50	−0.04	0.12	0.14	1

^*^p < 0.05.

^**^p < 0.01.

### 3.3 Source of negative emotions

College students had significantly higher negative emotions during COVID-19, which included higher anxiety and depression. In the context of the closure of schools, college students, as a particular group in the transition from students to social adults, were more likely to be affected by bad attitudes and emotions regarding the pandemic from people on the Internet or around them. This could lead to psychological panic as they were in high-risk areas or lacked materials. Some college students also reduced the scope of free activities due to the closure and lack of entertainment. This was consistent with our hypothesis. Table 4 presents the sources of negative emotions selected by college students according to their actual conditions during the closure period.

**Table 4:** Sources of negative emotions.

		**Highly inconsistent**	**Relatively inconsistent**	**Difficult to confirm**	**Relatively match**	**Highly match**
Presence of potential threats	I have a fear of the spread of pandemic diseases, *N* (%)	80 (20.1)	120 (30.1)	58 (14.5)	123 (30.8)	18 (4.5)
		Material shortage, *N* (%)	117 (29.3)	150 (37.6)	57 (14.3)	56 (14.0)	19 (4.8)
Pressure of public opinion	Bad emotions passed by people around me make me uneasy, *N* (%)	90 (22.6)	106 (26.6)	66 (16.5)	108 (27.1)	29 (7.3)
		Bad emotions transmitted by the Internet make me uneasy, *N* (%)	99 (24.8)	98 (24.6)	63 (15.8)	114 (28.6)	25 (6.3)
Pressure of self-growth	I have academic pressure, *N* (%)	48 (12)	50 (12.5)	44 (11)	185 (46.4)	72 (18.0)
		I am under pressure to seek employment, *N* (%)	69 (17.3)	49 (12.3)	54 (13.5)	146 (36.6)	81 (20.3)
Narrowing the scope of social activities	Influence of school closure management measures, *N* (%)	47 (11.8)	68 (17)	100 (25.1)	138 (34.6)	46 (11.5)
		Lack of social contact, *N* (%)	79 (19.8)	92 (23.1)	53 (13.3)	130 (32.6)	45 (11.3)
		Lack of social freedom and small scope of activities, *N* (%)	47 (11.8)	57 (14.3)	39 (9.8)	174 (43.6)	82 (20.6)
		Lack of entertainment, *N* (%)	58 (14.5)	68 (17)	37 (9.3)	165 (41.4)	71 (17.8)

As shown in Table 5, the correlation analysis revealed a significant positive correlation between anxiety, depression, the existence of potential threats, the pressure of public opinion, the pressure of self-growth, and the narrowing of the scope of social activities [*r*_(397)_ = 0.451–0.793, *p* < 0.01]. This indicated that the existence of potential threats, the pressure of public opinion, the pressure of self-growth, and the narrowing of the scope of social activities had a significant impact on college students' anxiety and depression.

**Table 5:** Descriptive statistics and correlation analysis of the sources of anxiety and depression among college students.

	***M ±SD***	**1**	**2**	**3**	**4**	**5**	**6**
1. Depression	0.55 ± 0.53	1					
2. Anxiety	0.79 ± 0.72	0.793^**^	1				
3. Presence of potential threats	2.49 ± 0.95	0.462^**^	0.453^**^	1			
4. The pressure of public opinion	2.68 ± 1.20	0.498^**^	0.549^**^	0.601^**^	1		
5. Pressure of self-growth	3.38 ± 1.16	0.453^**^	0.526^**^	0.474^**^	0.528^**^	1	
6. Narrowing the scope of social activities	3.22 ± 1.04	0.451^**^	0.540^**^	0.558^**^	0.528^**^	0.551^**^	1

^**^p < 0.01.

Our research summarized the sources of negative emotions into three types. First, individuals could feel uneasy regarding whether the pandemic would affect their physical health and worried about their future, such as academic and employment issues after graduation. Second, the environmental impact could be influenced by a panicked atmosphere conveyed by people and the Internet, which made it easier to fall into a herd mentality due to social public opinion. Third, the comprehensive influence of behavior, environment, and individual internal factors could result in negative emotions. In a closed environment, individuals lacked social activities and entertainment methods, which made it difficult to relieve stress and more likely to exacerbate negative emotions. There were various sources of negative emotions among college students during the COVID-19 pandemic. These should be considered from multiple perspectives so that adequate measures can be taken to help students cope with and alleviate these emotions. This study provided a specific analysis of the sources that caused negative emotions, such as anxiety or depression, in students.

#### 3.3.1 Existence of potential threats

Realistic anxiety represents an emotional response to potential challenges or threats to reality. This emotional response was adapted to the reality of threats, a general response when a person faced events or situations beyond their control (35). The characteristic was that the intensity of anxiety was consistent with the degree of the real threat and disappeared with the disappearance of the real danger (35). With the nationwide and global spread of the pandemic, college students in middle- and high-risk areas could face a significant potential disease threat. Conversely, the shortage of materials also caused college students' psychological panic and, thus, affected their health.

#### 3.3.2 Pressure of public opinion

The rapid development of the network has led to an era of information. Ways of transmitting information have been diversified, and the speed of transmission has increased. Increasing information can be obtained from different sources of knowledge. During the outbreak of COVID-19, in addition to obtaining relevant information through the news media, people could also obtain information through social media, mobile media, mobile platforms, and other channels. Therefore, negative emotions transmitted by people easily increase and cause anxiety, depression, and other negative emotions. Furthermore, rumors and sensational information could also cause or aggravate anxiety and stress.

#### 3.3.3 Pressure of self-growth

Influenced by the pandemic, colleges and universities adopted online teaching. Uncertainty of the situation, changes in teaching methods, and students' maladjustment could easily impact students' anxiety, impetuosity, worry, and other negative emotions. Our results showed that 64.4% of college students had academic pressure. In addition to completing routine study tasks, some college students faced other heavy tasks, such as preparing for the postgraduate entrance examination and obtaining CET-4/CET-6 or various qualification certificates. In addition to their academic performance, students also paid attention to employment. Almost 56.9% had employment/job-hunting pressure.

#### 3.3.4 Narrowing social activities' range

School closure measures cause physical space restrictions among college students. Students were strictly confined to a limited range, and their recreation and social activities were significantly reduced. Nearly 75.4% had free activities on campus, and 7.0% were required to stay in dormitories. Activity spaces were also smaller than before. Students' interpersonal communication and interaction opportunities were reduced, and a lack of social support increased anxious or depressed feelings. College students also lost their usual diversified learning environment; their monotonous lives made them lack perception and experience. Furthermore, their inner anxiety led to large emotional fluctuations, which resulted in increased anxiety and depression.

In addition, 13.5% of college students' negative emotions came from the potential threat of the pandemic. On average, 17.3% reported negative emotions from the influence of destructive emotions on the Internet or those around them. Furthermore, 30.3% reported negative emotions from the pressure of their growth, such as the pressure of academics or seeking jobs. In addition, 26.8% reported negative emotions from the reduced scope of social activities. Hence, college students' negative emotions were affected in all aspects and cannot be ignored.

### 3.4 Strategies for regulating negative emotions

College students undertook various measures to manage and alleviate their negative emotions. They usually adopted cognitive changes and implemented positive behaviors and coping strategies to manage their feelings better. Table 6 presents the self-regulation strategies for negative emotions reported by college students according to their own experiences. Table 6, 1, 2, 3, and 4 represent “I regulate my anxiety and depression through learning, reading, and collecting information about the pandemic situation,” “I relax appropriately through sports/online games/listening to music/watching videos,” “I choose remote psychological counseling (telephone or Internet) to adjust,” and “I communicate with my family and friends over the phone or on the Internet for help,” respectively.

**Table 6:** Ways of regulating negative emotions.

	***M ±SD***	**Highly inconsistent**	**Relatively inconsistent**	**Difficult to confirm**	**Relatively match**	**Highly match**
1. I regulate my anxiety and depression by learning, reading, and collecting information on the pandemic, *N* (%)	3.27 ± 1.13	32 (8.0)	81 (20.3)	70 (17.5)	179 (44.9)	37 (9.3)
2. Scope of social activities is reduced. I can relax properly through sports/online games/listening to music/watching videos, etc., *N* (%)	4.03 ± 0.90	10 (2.5)	22 (5.5)	29 (7.3)	222 (55.6)	116 (29.1)
3. I choose remote psychological consultation (telephone or Internet) to adjust, *N* (%)	2.25 ± 1.21	138 (34.6)	123 (30.8)	54 (13.5)	68 (17.0)	16 (4.0)
4. I communicate and talk with my family and friends over the phone or on the Internet for help, *N* (%)	3.65 ± 1.14	33 (8.3)	39 (9.8)	37 (9.3)	215 (53.9)	75 (18.8)

#### 3.4.1 Get scientific information through the right channels and staying away from harmful information

College students should use scientific methods to obtain relevant information from formal official websites or channels, master the necessary and correct pandemic-related knowledge, avoid contact with lousy information with strong feelings from the source, and prevent the psychological effects of information overload. In a psychological panic, people should cut off contact with lousy information and avoid being affected by the destructive emotions of people and those on the Internet. In our study, 54.3% of respondents adjusted their negative emotions by learning, reading, and collecting information. According to the ABC theory, the direct cause of negative emotions in individuals was not the event itself but the cognitive evaluation of the event (36). Efforts to change cognitive styles and establish an optimistic attitude could help reduce negative emotions and alleviate anxiety and depression among college students. When the network is mostly related to negative emotions, students should pay attention to the identification and screening of this information. In addition, they should spread positive energy to their friends and relatives around the network.

#### 3.4.2 Get relaxation and reduce the level of anxiety and depression

Positive behavior and coping strategies could help college students alleviate negative emotions. During the COVID-19 pandemic, although college students' lives and learning styles underwent tremendous changes, they had more time to enjoy life and plan their own learning time (37). During this period, many funny shows and comedies emerged on network platforms. In our study, 84.7% chose to adjust their negative emotions through sports/online games/listening to music/watching videos, and other appropriate relaxation methods to relax and actively reduce their anxiety and depression levels. This result shows that it was a benign way to regulate negative emotions and reduce stress appropriately.

#### 3.4.3 Seek psychological counseling from professionals

Many official psychological counseling institutions opened hotlines with the confidentiality of visitors' information and basic security guarantees. This method effectively avoided direct contact with professional psychological consultants and could provide professional guidance for visitors. College students could also seek help from professional counselors by making an appointment with the school's psychological counseling room. The professional could provide psychological counseling. When necessary, music therapy, muscle relaxation training, or biological feedback enabled students to fight against and quickly and effectively eliminate general psychological and emotional stress symptoms. In our study, 21% of college students chose remote psychological counseling (telephone or Internet) to regulate their negative emotions. Simple psychological catharsis methods could be applied to relieve mild anxiety and depression symptoms. In severe cases, it was essential to seek professional help.

#### 3.4.4 Actively establish interpersonal communication

Maintaining regular interpersonal connections is an essential way for college students to obtain emotional support and avoid loneliness, depression, and other destructive emotions. In our study, 72.7% of college students chose to communicate and talk with their family and friends on the phone or the Internet and sought help to regulate their negative emotions. College students should establish good interpersonal relationships with their parents, teachers, and classmates. A positive communication circle was conducive to developing college students' mental health. Interpersonal communication was the first step to alleviating negative emotions, which could make students feel warm and help combat the impact of destructive emotions. Interpersonal care could provide spiritual enlightenment, obtain emotional support, harvest warmth and strength, enhance positive emotions, and increase confidence.

## 4 Discussion

The incidence of anxiety and depression among 399 undergraduates from seven colleges and universities was 23.3 and 20.1%, respectively, which was consistent with rates reported in previous studies (38, 39). The incidence of negative emotions, such as anxiety and depression, dramatically increased among college students during the COVID-19 pandemic. This research has some suggestions to improve college students' negative emotions.

### 4.1 Sex differences in negative emotions

Recent studies found that the detection rate and degree of negative emotions, such as depression and anxiety, were higher among female college students than among male college students (40). Figure 1 presents the proportion of anxiety and depression among college students by sex. Levels of anxiety and depression among female college students were higher than among male college students. Female college students were more likely to have negative emotions, and their psychological status was more affected. This was consistent with previous studies. Li et al. found that women were greatly affected by the pandemic, and their negative emotions, anxiety, and depression levels were higher than those of men (41). The reason was that the physiological differences between women and men (such as congenital vulnerability, hormone and cortisol levels, etc.) were reflected in their emotions and behaviors. Women had more vital empathy than men (42), which made them more vulnerable to pandemic-related events and caused emotional fluctuations. In major public crisis events, women were usually more alert. Furthermore, female college students' stress susceptibility could induce negative emotional states, which lead to emotional problems such as anxiety (43). Moreover, traditional gender cognition affected their attitudes and behaviors toward life events. Men were more likely to adopt externalized behaviors to dispel emotions, while women generally showed internalized emotional fluctuations. Hence, they were more likely to have depressive symptoms (44).

### 4.2 Grade differences in negative emotion

Previous studies found differences in the detection rate of depressive symptoms in different grades. Furthermore, the higher the grade, the higher the detection rate of depressive symptoms (40). The degree of depression among college students in different grades was different. The rate of moderate and severe depression among senior students was higher, which could be related to their anxiety about facing new life choices (45). The incidence of depression and anxiety among senior college students was significantly higher than that of junior and middle-grade students (46). Levels of anxiety and depression were the highest among senior students. Furthermore, the level of depression was significantly different by grade. Further analysis found a significant difference in the level of depression between freshmen and sophomores. In addition, a substantial difference was also observed in the level of depression between freshmen and seniors, which was consistent with previous studies. The reason was that freshmen had just entered the university and were still in the adaptation period.

### 4.3 Strategies and suggestions for improving college students' negative emotions

#### 4.3.1 Engage in positive self-talk

A psychological suggestion was to influence the psychology and behavior of others or oneself implicitly and indirectly, which often led others to take certain actions unconsciously, uncritically, or specific views or beliefs, respectively (47). It affected human psychology, behavior, and physiological function (36). Positive psychological hints could provide people with infinite faith and hope and, thus, mobilize people's endless potential and play into their internal strength. Negative psychological hints could lead to anxiety and fear of life, inhibit intelligence and ability, and damage physical health (48). Many psychological experiments proved that positive self-suggestion could eliminate tension and anxiety and establish an optimistic psychological state. Therefore, individuals should provide themselves with daily positive psychological hints and avoid negative hints (36).

#### 4.3.2 Meditative mindfulness training

Mindfulness meditation is a group of meditation practices with mindfulness technology at its core. Mindfulness meditation training emphasizes being fully engaged in the present and paying attention to the immediate experience of the present. It was a free and open conscious attention process that advocated actively paying attention to painful experiences with an open and receptive attitude (48). Researchers from the University of Quebec in Canada suggested that practicing mindfulness meditation (such as body scanning) was feasible to reduce the possibility of experiencing such a crisis (49). Meditation gradually affects one's mental health. It developed one's intelligence and also relieved psychological pressure, improved individual attention, and promoted the ability to adapt to society through emotional influence.

#### 4.3.3 Learn to pour out and ask for help

Learning to talk was an effective way for students to face difficulties in life, overcome the entanglement of negative emotions, and reduce psychological difficulties. It was also an effective way to help students find their lost confidence, restore good interpersonal relationships, enhance their resilience, help them understand themselves, develop their body and mind, and have a positive and healthy lifestyle.

## 5 Conclusion

This study conducted a stratified cluster sampling questionnaire to explore the source of college students' negative emotions and strategies of self-emotion regulation during the school closure period of the COVID-19 pandemic. The prevalence of anxiety and depression in college students was 23.3 and 20.1%, respectively. Furthermore, the level of anxiety and depression was higher among women than among men. The grade difference was significant in negative emotions, which showed that senior students had higher levels of anxiety and depression than freshmen. In addition, anxiety and depression came from the pressure of their growth and the narrowing of the scope of social activities. Relaxing properly through entertainment and communicating and talking to family and friends were the most popular ways to regulate negative emotions among college students. Our findings suggested that colleges and universities should pay attention to students' mental health when conducting pandemic prevention and control measures. Differences exist in the sources of negative emotions among college students. Furthermore, individuals did not fully master the self-regulation methods for negative emotions. College students should face and grasp the source of negative emotions, learn to use psychological methods and self-psychological evaluation, master self-regulation strategies for negative emotions, or actively seek professional help. Furthermore, they should aim to reduce anxiety and depression, weaken the negative impact of these emotions, enhance psychological flexibility, and undertake positive coping methods to solve problems.

### 5.1 Limitations

This study has some limitations. First, college students were a group highly concerned by society. Due to the limitations of data and manpower, seven universities, which included Nantong University, Ningbo University, and Wuhan University of Light Industry, were selected. Follow-up research should expand on the number of samples and scope of the research. Second, this study was a cross-sectional design, which could only present static development results.

### 5.2 Future research and recommendations

College students were more fragile than adults when faced with mental pressure, such as the COVID-19 pandemic and other public health crises. Future studies should focus on college students' mental health and add new data from questionnaires and interviews. Based on the relevant research literature, future studies should use new ideas and findings, conduct accurate experiments, and conduct larger studies with data. Additional results can be beneficial toward easing college students' mental pressure and helping them be beneficial to society.

## Data availability statement

The datasets used and/or analyzed during the current study are available from the corresponding author on reasonable request. Requests to access the datasets should be directed to MP, ntupmf@163.com.

## Ethics statement

The studies involving humans were approved by Nantong University Ethics Committee. The studies were conducted in accordance with the local legislation and institutional requirements. The participants provided their written informed consent to participate in this study. Written informed consent was obtained from the individual(s) for the publication of any potentially identifiable images or data included in this article.

## Author contributions

HF: Conceptualization, Data curation, Formal analysis, Funding acquisition, Investigation, Methodology, Project administration, Resources, Software, Supervision, Validation, Writing – original draft, Writing – review & editing. MP: Data curation, Investigation, Methodology, Resources, Software, Writing – original draft. ML: Funding acquisition, Project administration, Resources, Software, Supervision, Writing – review & editing.

## References

[1] Lin L Wang X Yuan J Chen Y. Teaching practice of ideological and political education in the course of introduction immunology: take the development of vaccines as a teaching case. J Heilongjiang Voc Inst Ecol Eng. (2021) 34:148–51. 10.3969/j.issn.1674-6341.2021.04.037

[2] Taylor S. The Psychology of Pandemics: Preparing for the Next Global Infectious Disease Outbreak. Newcastle upon Tyne: Cambridge Scholars Publishing (2019).

[3] Orrù G Ciacchini R Gemignani A Conversano C. Psychological intervention measures during the COVID-19 pandemic. Clin Neuropsychiatry. (2020) 17:76–9. 10.3390/ijerph17186688 34908972 PMC8629089

[4] Hossain MM Tasnim S Sultana A Faizah F Mazumder H Zou L. Epidemiology of mental health problems in COVID-19: a review. F1000Research. (2020) 9:636. 10.12688/f1000research.24457.1 33093946 PMC7549174

[5] Di Giuseppe M Zilcha-Mano S Prout TA Perry JC Orrù G Conversano C. The psychological impact of coronavirus disease 2019 among Italians during the first week of lockdown. Front Psychiatry. (2020) 11:576597. 10.3389/fpsyt.2020.576597 33192713 PMC7554332

[6] Brooks SK Webster RK Smith LE Woodland L Wessely S Greenberg N. The psychological impact of quarantine and how to reduce it: rapid review of the evidence. Lancet. (2020) 395:912–20. 10.1016/S0140-6736(20)30460-8 32112714 PMC7158942

[7] Elharake JA Akbar F Malik AA Gilliam W Omer SB. Mental health impact of COVID-19 among children and college students: a systematic review. Child Psychiatry Hum Dev. (2022) 54:913–25. 10.1007/s10578-021-01297-1 35013847 PMC8747859

[8] Hiremath P Kowshik CSS Manjunath M Shettar M. COVID-19: impact of lock-down on mental health and tips to overcome. Asian J Psychiatry. (2020) 51:102088. 10.1016/j.ajp.2020.102088 32302964 PMC7151434

[9] Ahmad N Seymour RG. Defining entrepreneurial activity: definitions supporting frameworks for data collection. OECD Stat Work Pap. (2008) 1:1–18. 10.1787/243164686763

[10] Erum S Muhammad NT Wali Muhammad K Mohsen B Shahid R. Chapter 8: the COVID-19 pandemic overlaps entrepreneurial activities and triggered new challenges: a review study. In: New Teaching Resources for Management in a Globalised World Managing Human Resources in SMEs and Start-Ups (2022). pp. 155–82. 10.1142/9789811239212_0008

[11] Joshi M Brahmi MS. A mediation-based analysis of emotional intelligence effect on cognition and consumer decision-making. Int J Publ Sect Perform Manag. (2023) 12:223–51. 10.1504/IJPSPM.2023.132251

[12] Arabiun A Dehkordi AM Hosseini E Brahmi M. A framework for strategic analysis in dynamic and complex environments. Explor Bus Ecosyst Innov Capacity Build Glob Econ. (2023) 4:ch002. 10.4018/978-1-6684-6766-4.ch002

[13] Wang C Cheng Z Yue XG McAleer M. Risk management of COVID-19 by universities in China. J Risk Financ Manag. (2020) 13:36. 10.3390/jrfm13020036

[14] Xiang YT Yang Y Li W Zhang L Zhang Q Cheung T. Timely mental health care for the 2019 novel coronavirus outbreak is urgently needed. Lancet Psychiatry (2020) 7:228–9. 10.1016/S2215-0366(20)30046-8 32032543 PMC7128153

[15] Martino G Caputo A Bellone F Quattropani MC Vicario CM. Going beyond the visible in type 2 diabetes mellitus: defense mechanisms and their associations with depression and health-related quality of life. Front Psychol. (2020) 11:267. 10.3389/fpsyg.2020.00267 32174865 PMC7054284

[16] Rottenberg J Gross JJ. Emotion and emotion regulation: a map for psychotherapy researchers. Clin Psychol. (2007) 14:323–28. 10.1111/j.1468-2850.2007.00093.x

[17] Gao F. Emotional Control Technique. Beijing: Institute of Technology Press (2010).

[18] Chen J. A Study on the Correlation Between Self-Emotional Management and Academic Performance of High School Students (master's thesis). Qinghai Normal University, Xining, China (2018). Available online at: https://kns.cnki.net/KCMS/detail/detail.aspx?dbname=CMFD201901&filename=1018241977.nh (accessed February 22, 2023).

[19] Nahal S Bryan G William T Bridget I. College students mental health challenges: concerns and considerations in the COVID-19 pandemic. J Coll Stud Psychother. (2023) 37:39–51. 10.1080/87568225.2021.1890298

[20] Hwang EH Kim KH. Relationship between optimism, emotional intelligence, and academic resilience of nursing students: the mediating effect of self-directed learning competency. Front Public Health. (2023) 11:52689. 10.3389/fpubh.2023.1182689 37275498 PMC10234118

[21] Shek D Zhu X Dou D. A mental health survey and promoting psychological well-being in university students under COVID-19. J Altern Med Res. (2023) 15:9–172.

[22] Liu YH Zhang YX Ai L Sang XH Wan H. Self-efficacy and emotional intelligence in clinical nurses related to negative psychology and burnout. Psychol Res Behav Manag. (2023) 26:3333–45. 10.2147/PRBM.S417729 37650114 PMC10463736

[23] Chen YL. Emotional stability and mental health in art vocational and technical college students during epidemic prevention and control. Psychol Res Behav Manag. (2023) 26:2857–67. 10.2147/PRBM.S417243 37525850 PMC10387273

[24] Lopes PN Salovey P Côté S Beers M Petty RE. Emotion regulation abilities and the quality of social interaction. Emotion. (2005) 5:113–18. 10.1037/1528-3542.5.1.113 15755224

[25] Nesse RM. Evolutionary explanations of emotions. Hum Nat. (1990) 1:261–89. 10.1007/BF02733986 24222085

[26] Ding XF. Distance Education. 2nd ed. Beijing: Beijing Normal University Press (2009).

[27] Wang N Ma J. Analysis of factors influencing aggressive behavior among netizens in hot public opinion events. Intell Exp. (2023) 2:46–55.

[28] Kirby P Mattia DD. A rational approach to emotional management. Train Dev J. (1991) 45:67–70.

[29] Guo ZZ. A study on the relationship between adolescent self-forgiveness, emotional management, and mental health (master's thesis). Southwest Jiaotong University, Chengdu, China (2021).

[30] Shi X Luo H Fang R Qi J Ping S Zhang C. Status and influencing factors of anxiety and depression in college students during the outbreak of novel coronavirus pneumonia in Hubei province. Henan J Prev Med. (2021) 32:657–61. 10.13515/j.cnki.hnjpm.1006-8414.2021.09.004

[31] Clark LA Watson D. Tripartite model of anxiety and depression: psychometric evidence and taxonomic implications. J Abnorm Psychol. (1991) 100:316–36. 10.1037//0021-843X.100.3.316 1918611

[32] Lovibond SH Lovibond PF. Manual for the depression. Anxiety Stress Scales (1995). 10.1016/0005-7967(94)00075-u 7726811

[33] Shan X Qiu J Wang B Dang Y Lu T Zheng Y. Big data management and analytics in scientific programming. Sci Program. (2020) 2020:5060635. 10.1155/2020/5060635

[34] Gong X Xie X Xu R Luo Y. Psychometric properties of the Chinese versions of DASS-21 in Chinese college students. Chinese J Clin Psychol. (2010) 18:443–6. 10.16128/j.cnki.1005-3611.2010.04.020

[35] Wang SS. Interpreting “Anxiety Disorder”. Psychologist. (2016) 32.

[36] Ellis A. The revised ABCs of rational-emotive therapy (RET). J Ration-Emot Cogn-Behav Ther. (1991) 3:139–72. 10.1007/BF01061227

[37] Wang SJ Li DX Ma LY. An Empirical Study on the Evaluation of the Professional Ability of College Counselors – A Case Study of Zhejiang University. J Wuhan Vocat Tech College. (2017) 4:45–51.

[38] Chang J Yuan Y Wang D. Mental health status and its influencing factors among college students during the epidemic of COVID-19. Nan Fang Yi Ke Da Xue Bao. (2020) 40:171–76. 10.12122/j.issn.1673-4254.2020.02.06 32376528 PMC7086131

[39] Gao D Zhang H. Analysis on the status of anxiety and depression of college students in a university. Med Res Educ. (2019) 36:1–46. 10.3969/j.issn.1674-490X.2019.04.008

[40] Huang Y Ma J. Scheme design of group art therapy to promote the mental health of female college students. Beijing Educ. (2022) 96:87–90. 10.12014/j.issn.1002-0772.2020.24.12

[41] Li X Luo J Gao W Yuan J. Research on negative emotions, coping style, self-esteem, and interpersonal relationships of college students with left-behind experience. Chin J Clin Psychol. (2009) 17:20–622. 10.16128/j.cnki.1005-3611.2009.05.004

[42] Yan Z Su Y. Gender difference in empathy: the evidence from a meta-analysis. Psychol Dev Educ. (2018) 34:129–36. 10.16187/j.cnki.issn1001-4918.2018.02.01

[43] Guo L Xu P Yao F Zhang F Qi L Yang F. The effect of acute stress disorder on negative emotions in Chinese public during the NCP epidemic: the moderating effect of social support. J Southwest Univ. (2020) 42:21–30. 10.13718/j.cnki.xdzk.2020.05.003

[44] Qiao S. Study on the Prevalence and Influencing Factors of Depressive Symptoms Among College Students in Jilin Province (master's thesis), Jilin University, Changchun, China (2022). Available online at: https://kns.cnki.net/KCMS/detail/detail.aspx?dbname=CMFD202301&filename=1022528868.nh (accessed March 2, 2023).

[45] Zheng S Tong Q Zheng A. Investigation on depression and anxiety of college students and analysis of related factors. Chongqing Med. (2016) 45:2835–37. 10.3969/j.issn.1671-8348.2016.20.032

[46] Chen X Deng G. Use positive self-suggestion to stimulate infinite positive energy: design a psychological counseling activity class. J Moral Educ Prim Second Sch. (2013) 6:47–9.

[47] Wu J. Research on the cultivation of athletes' positive psychology and training effect. J Xianning Univ. (2011) 31:148–49. 10.16751/j.cnki.hbkj.2011.10.064

[48] Wang Q Dong L Cheng J Liu Y. Effect of mindfulness-based music therapy on sleep-wake behavior in patients with cerebral hemorrhage and sleep disorders. J Nurs. (2018) 25:5–8. 10.16460/j.issn1008-9969.2018.16.005

[49] Xing Q Xing X. Effect of mindfulness meditation training on anxiety and depression of AIDS patients. Medical For. (2022) 26:142–44. 10.19435/j.1672-1721.2022.22.04531511223

